# Supplementary material for: Effects of grazing intensity and the use of veterinary medical products on dung beetle biodiversity in the sub-mountainous landscape of Central Italy
Source: PeerJ. 2017 Jan 12;5:e2780. doi: 10.7717/peerj.2780 (PMC5237365; doi:10.7717/peerj.2780)
Supplement: Supplemental Information 1 — Number of individuals of dung beetle species collected from each treatment of the two factors. For each treatment, inventory completeness was also reported according to the coverage-based rarefaction and extrapolation estimators. LG, low grazing; MG, moderate grazing; ECO, VMP-free; VMP, with the use of VMPs. [file peerj-05-2780-s001.docx]

Supplementary Material S1. Raw data of dung beetles collected in all sites studied.

| **Species** | **ECO** | **VMP** | **LG** | **MG** | **Total** |
| --- | --- | --- | --- | --- | --- |
| *Acanthobodilus immundus* (Creutzer, 1799) | 21 | 2 | 4 | 19 | 23 |
| *Acrossus luridus* (Fabricius, 1775) | 217 | 9 | 170 | 56 | 226 |
| *Acrossus rufipes* (Linnaeus, 1758) | 0 | 4 | 0 | 4 | 4 |
| *Agrilinus constans* (Duftschmid, 1805) | 1 | 0 | 0 | 1 | 1 |
| *Agrilinus convexus* (Erichson, 1848) | 23 | 0 | 0 | 23 | 23 |
| *Aphodius coniugatus* (Panzer, 1795) | 4 | 2 | 5 | 1 | 6 |
| *Aphodius fimetarius* (Linnaeus, 1758) | 124 | 13 | 119 | 18 | 137 |
| *Aphodius foetidus* (Herbst, 1783) | 9 | 0 | 3 | 6 | 9 |
| *Biralus mahunkaorum* (Ádám, 1983) | 0 | 1 | 1 | 0 | 1 |
| *Bodilopsis rufa* (Moll, 1782) | 42 | 779 | 21 | 800 | 821 |
| *Bodiloides ictericus* (Laicharting, 1781) | 11 | 0 | 0 | 11 | 11 |
| *Calamosternus granarius* (Linnaeus, 1767) | 20 | 2 | 0 | 22 | 22 |
| *Calamosternus mayeri* (Pilleri, 1953) | 1 | 0 | 1 | 0 | 1 |
| *Chilothorax conspurcatus* (Linnaeus, 1758) | 749 | 32 | 732 | 49 | 781 |
| *Chilothorax lineolatus* (Illiger, 1803) | 3 | 0 | 2 | 1 | 3 |
| *Chilothorax paykulli* (Bedel, 1907) | 53 | 0 | 0 | 53 | 53 |
| *Colobopterus erraticus* (Linnaeus, 1758) | 1005 | 1964 | 1706 | 1263 | 2969 |
| *Coprimorphus scrutator* (Herbst, 1789) | 6 | 167 | 3 | 170 | 173 |
| *Esymus merdarius* (Fabricius, 1775) | 103 | 3 | 102 | 4 | 106 |
| *Esymus pusillus* (Herbst, 1789) | 19 | 8 | 14 | 13 | 27 |
| *Euorodalus paracoenosus* (Balthasar & Hrubant, 1960) | 2 | 0 | 0 | 2 | 2 |
| *Labarrus lividus* (Olivier, 1789) | 219 | 310 | 125 | 404 | 529 |
| *Limarus zenkeri* (Germar, 1813) | 2 | 0 | 0 | 2 | 2 |
| *Loraphodius suarius* (Faldermann, 1835) | 47 | 5 | 34 | 18 | 52 |
| *Melinopterus consputus* (Creutzer, 1799) | 94951 | 6449 | 98709 | 2691 | 101400 |
| *Melinopterus prodromus* (Brahm, 1790) | 7289 | 20 | 1135 | 6174 | 7309 |
| *Melinopterus reyi* (Reitter, 1892) | 16 | 0 | 0 | 16 | 16 |
| *Melinopterus stolzi* (Reitter, 1906) | 2 | 0 | 0 | 2 | 2 |
| *Nialus varians* (Duftschmid, 1805) | 9 | 0 | 0 | 9 | 9 |
| *Nimbus contaminatus* (Herbst, 1783) | 464 | 341 | 470 | 335 | 805 |
| *Nimbus johnsoni* (Baraud, 1976) | 20 | 1 | 7 | 14 | 21 |
| *Nimbus obliteratus* (Panzer, 1823) | 2309 | 260 | 2170 | 399 | 2569 |
| *Otophorus haemorrhoidalis* (Linnaeus, 1758) | 21 | 39 | 0 | 60 | 60 |
| *Phalacronothus biguttatus* (Germar, 1824) | 4 | 0 | 1 | 3 | 4 |
| *Planolinus fasciatus* (Olivier, 1789) | 1 | 0 | 1 | 0 | 1 |
| *Sigorus porcus* (Fabricius, 1792) | 234 | 102 | 254 | 82 | 336 |
| *Teuchestes fossor* (Linnaeus, 1758) | 0 | 11 | 0 | 11 | 11 |
| *Trichonotulus scrofa* (Fabricius, 1787) | 192 | 54 | 192 | 54 | 246 |
| *Bubas bison* (Linnaeus, 1767) | 103 | 3 | 14 | 92 | 106 |
| *Caccobius schreberi* (Linnaeus, 1767) | 5 | 8 | 2 | 11 | 13 |
| *Copris lunaris* (Linnaeus, 1758) | 15 | 8 | 3 | 20 | 23 |
| *Euoniticellus fulvus* (Goeze, 1777) | 4955 | 2401 | 4359 | 2997 | 7356 |
| *Onthophagus coenobita* (Herbst, 1783) | 168 | 16 | 36 | 148 | 184 |
| *Onthophagus fracticornis* (Preyssler, 1790) | 3157 | 5881 | 1418 | 7620 | 9038 |
| *Onthophagus grossepunctatus* Reitter, 1905 | 110 | 0 | 0 | 110 | 110 |
| *Onthophagus illyricus* (Scopoli, 1763) | 1 | 1 | 1 | 1 | 2 |
| *Onthophagus joannae* Goljan, 1953 | 26 | 7 | 31 | 2 | 33 |
| *Onthophagus lemur* (Fabricius, 1781) | 324 | 66 | 74 | 316 | 390 |
| *Onthophagus medius* (Kugelann, 1792) | 10151 | 666 | 10216 | 601 | 10817 |
| *Onthophagus opacicollis* Reitter, 1892 | 36 | 0 | 12 | 24 | 36 |
| *Onthophagus ruficapillus* Brullé, 1832 | 178 | 6 | 7 | 177 | 184 |
| *Onthophagus taurus* (Schreber, 1759) | 710 | 81 | 69 | 722 | 791 |
| *Onthophagus verticicornis* (Laicharting, 1781) | 122 | 165 | 149 | 138 | 287 |
| *Sisyphus schaefferi* (Linnaeus, 1758) | 10 | 9 | 11 | 8 | 19 |
| *Sericotrupes niger* (Marsham, 1802) | 229 | 24 | 75 | 178 | 253 |
| *Geotrupes spiniger* Marsham, 1802 | 123 | 125 | 153 | 95 | 248 |
| *Trypocopris vernalis apenninicus* Mariani, 1958 | 0 | 7 | 0 | 7 | 7 |
| Total species (S) | 53 | 41 | 42 | 54 | 57 |
| Total individuals (N) | 128616 | 20052 | 122611 | 26057 | 148668 |
| Sampling coberture (%) | 99 | 99 | 100 | 99 |  |
