# Supplementary material for: Effects of grazing intensity and the use of veterinary medical products on dung beetle biodiversity in the sub-mountainous landscape of Central Italy
Source: PeerJ. 2017 Jan 12;5:e2780. doi: 10.7717/peerj.2780 (PMC5237365; doi:10.7717/peerj.2780)
Supplement: Supplemental Information 2 [file peerj-05-2780-s002.docx]

Supplementary Material S2

*Dung beetle identification*

Scarabaeinae and Geotrupinae subfamilies have been identified according to Baraud (1992). Aphodiinae subfamily were identified following Dellacasa and Dellacasa (2006). *Biralus mahunkaorum* (Ádám, 1983), *Onthophagus medius* (Kugelann, 1792) and *Aphodius fimetarius* (Linnaeus, 1758) were identified following the work, respectively, of Rössner and Fery (2014), Rössner et al. (2010) and Miraldo et al. (2014), respectively. The species of the *ovatus* group (i.e. *Onthophagus ruficapillus* Brullé, 1832, *Onthophagus joannae* Goljan, 1953, *Onthophagus grossepunctatus* Reitter, 1905) were identified using the genitalia characters proposed by Martin-Piera and Zunino (1986) based on the work of Binaghi et al. (1969).

*Alpha diversity*

The Hill numbers’ family diversity are measures parameterized by the order *q*. The *q* parameter determines the sensitivity of the index with respect to rare or abundant species. For example, the diversity of *q* = 0 is completely insensitive to species abundance and so corresponds to species richness; for *q* = 1, species are weighted proportionally to their relative abundance and correspond to the Shannon entropy exponential; for *q* = 2 the index is disproportionately sensitive to common species and corresponds to the reciprocal of the Simpson index (Jost 2006, 2007). The results of these indices are expressed in terms of the “effective number of species”, i.e. the number of equally abundant species needed to produce the same value of the diversity measure (Jost 2006, 2007). These measures conform to the replication principle introduced by Hill (1973).

*Dung beetle biomass and abundance*

Fewer than 10 specimens of the following species were measured (the figure in brackets is the number of specimens measured): *Calamosternus mayeri* (Pilleri, 1953) (1); *Limarus zenkeri* (Germar, 1813) (1); *Melinopterus stolzi* (Reitter, 1906) (1); *Nimbus johnsoni* (Baraud, 1976) (1); *Phalacronotus biguttatus* (Germar, 1824) (1); *Planolinus fasciatus* (Olivier, 1789) (1); *Trypocopris vernalis apenninicus* Mariani, 1958 (4); *Nialus varian*s (Duftschmid, 1805) (4); and *Amidorus thermicola* (Sturm, 1800) (7).

*Beta diversity*

Like the Hill numbers, *q* is the same parameter that we used for alpha diversity and *N* is the number of assemblages (sites). When *q* = 0, *C_0N_* is equivalent to the multiple community version of the classical Sørensen index; when *q* = 1, *C_1N_* corresponds to the multiple community version of the Horn homogeneity measure, and when *q* = 2, *C_2N_* is equivalent to the multiple community version of the Morisita-Horn similarity index (Chao et al. 2012). For the integer values of *q* between 2 and *N*, the overlap measures *C_qN_* have a simple statistical interpretation as the ratio of two probabilities *^q^Gp/^q^Gs*. The numerator is the probability that *q* randomly sampled individuals belong to the same species given that they did not all come from the same assemblage. The denominator is the probability that *q* randomly sampled individuals belong to the same species given that they are all drawn from the same assemblage. This interpretation shows the depth of the measure: when *q* = 2 only the pairwise similarity is considered, but when *q* = 3 the measure also takes into account species that are shared by three assemblages (Jost et al 2011). This measure ranges from 0, when all the assemblages are completely different in terms of species composition, to 1 when they are identical. In summary, the measure *C_qN_* quantifies the effective average overlap per community, i.e. the average percentage of overlapped species in an assemblage. Its inverse is an estimate of the beta diversity.

References

Baraud, J., 1992. Coléoptères Scarabaeoidea d’Europe. Faune de France. France et Régions Limitrophes. Vol. 78. Société Linnéenne de Lyon. Lyon, France, 856 pp.

Dellacasa, G., Dellacasa, M., 2006. Coleoptera Aphodiidae, Aphodiinae. Fauna d’Italia Vol. XLI, Coleoptera Aphodiidae Aphodiinae. Calderini de Il Sole 24 Ore. Milán. Italia. 484 pp.

Rössner, E., Fery, H., 2014. *Aphodius* (*Biralus*) *mahunkaorum* (Ádám, 1983) in Europa (Coleoptera: Scarabaeidae, Aphodiinae). Entomologische Zeitschrift, Schwanfeld, 124(2): 113-121.

Rössner, E., Schönfeld, J., Ahrens D., 2010. Onthophagus (Palaeonthophagus) medius (Kugelann, 1792) – a good western paleartic species in the Onthophagus vacca complex (Coleoptera: Scarabaeidae: Scarabaeinae: Onthophagini). Zootaza, 2629: 1-28.

Miraldo, A., Krell, F.-T., Smalén, M., Angus, R.B., Roslin T., 2014. Making the cryptic visible – resolving the species complex of *Aphodius fimetarius* (Linnaeus) and *Aphodius pedellus* (de Geer) (Coleoptera: Scarabaeidae) by three complementary methods. Systematic Entomology, 39: 531-547.

Martín-Piera, F., Zunino, M., 1986. Analisi sistematica, filogenetica e biogeografica di un gruppo di specie del sottogenere *Palaeonthophagus* Zunino, 1979 (Coleoptera, Scarabaeidea: genere *Onthophagus*): il gruppo *ovatus*. Bollettino del Museo regionale di Scienze naturali di Torino, 40(2): 413-467.

Binaghi, G., Dellacasa, G., Poggi, R., 1969. Nuovi caratteri diagnostici per la determinazione degli Onthophagus del gruppo ovatus (L.) e geonemia controllata delle specie Italiane del gruppo. Memorie della Società Entomologica Italiana, 48(IB): 29-46.

Jost, L., 2006. Entropy and diversity. Oikos, 113: 363–375.

Jost, L., 2007. Partitioning diversity into independent alpha and beta components. Ecology, 88: 2427-2439.

Hill, M.O. 1973. Diversity and evenness: a unifying notation and its consequences. Ecology, 54: 427-432.

Chao, A., Chiu C.-H., Hsieh T.C. 2012. Proposing a resolution to debates on diversity partitioning. Ecology, 93(9): 2037-2051.

Jost L., Chao, A, Chazdon, R. 2011. Compositional similarity and β (beta) diversity. In: Magurran, A. and McGill, B. (Eds.). Biological Diversity: Frontiers in Measurement and Assessment. Oxford University Press, pp. 66-84.
